# Cerebral perfusion pressure and risk of brain hypoxia in severe head injury: a prospective observational study

**DOI:** 10.1186/cc3822

**Published:** 2005-10-14

**Authors:** Antonio J Marín-Caballos, Francisco Murillo-Cabezas, Aurelio Cayuela-Domínguez, Jose M Domínguez-Roldán, M Dolores Rincón-Ferrari, Julio Valencia-Anguita, Juan M Flores-Cordero, M Angeles Muñoz-Sánchez

**Affiliations:** 1 Staff Physician, Servicio de Cuidados Críticos y Urgencias, Hospitales Universitarios Virgen Del Rocío, Avda Manuel Siurot s/n, Seville, 41013, Spain; 2 Department Head, Servicio de Cuidados Críticos y Urgencias, Hospitales Universitarios Virgen Del Rocío, Avda Manuel Siurot s/n, Seville, 41013, Spain; 3 Methodological Consultant, Unidad de Apoyo a la Investigación, Hospitales Universitarios Virgen Del Rocío, Avda Manuel Siurot s/n, Seville, 41013, Spain; 4 Staff Physician, Servicio de Cuidados Críticos y Urgencias, Hospitales Universitarios Virgen Del Rocío, Avda Manuel Siurot s/n, Seville, 41013, Spain; 5 Staff Physician, Servicio de Cuidados Críticos y Urgencias, Hospitales Universitarios Virgen Del Rocío, Avda Manuel Siurot s/n, Seville, 41013, Spain; 6 Staff Physician, Servicio de Neurocirugía, Hospitales Universitarios Virgen Del Rocío, Avda Manuel Siurot s/n, Seville, 41013, Spain; 7 Staff Physician, Servicio de Cuidados Críticos y Urgencias, Hospitales Universitarios Virgen Del Rocío, Avda Manuel Siurot s/n, Seville, 41013, Spain; 8 Staff Physician, Servicio de Cuidados Críticos y Urgencias, Hospitales Universitarios Virgen Del Rocío, Avda Manuel Siurot s/n, Seville, 41013, Spain

## Abstract

**Introduction:**

Higher and lower cerebral perfusion pressure (CPP) thresholds have been proposed to improve brain tissue oxygen pressure (PtiO_2_) and outcome. We study the distribution of hypoxic PtiO_2 _samples at different CPP thresholds, using prospective multimodality monitoring in patients with severe traumatic brain injury.

**Methods:**

This is a prospective observational study of 22 severely head injured patients admitted to a neurosurgical critical care unit from whom multimodality data was collected during standard management directed at improving intracranial pressure, CPP and PtiO_2_. Local PtiO_2 _was continuously measured in uninjured areas and snapshot samples were collected hourly and analyzed in relation to simultaneous CPP. Other variables that influence tissue oxygen availability, mainly arterial oxygen saturation, end tidal carbon dioxide, body temperature and effective hemoglobin, were also monitored to keep them stable in order to avoid non-ischemic hypoxia.

**Results:**

Our main results indicate that half of PtiO_2 _samples were at risk of hypoxia (defined by a PtiO_2 _equal to or less than 15 mmHg) when CPP was below 60 mmHg, and that this percentage decreased to 25% and 10% when CPP was between 60 and 70 mmHg and above 70 mmHg, respectively (p < 0.01).

**Conclusion:**

Our study indicates that the risk of brain tissue hypoxia in severely head injured patients could be really high when CPP is below the normally recommended threshold of 60 mmHg, is still elevated when CPP is slightly over it, but decreases at CPP values above it.

## Introduction

The cerebral perfusion pressure (CPP) threshold that assures adequate cerebral perfusion still remains controversial in patients with traumatic brain injury (TBI); both higher and lower CPP thresholds have been proposed to improve outcome and brain tissue oxygen pressure (PtiO_2_). Several retrospective reports of outcomes related to CPP observed better results when CPP was > 80 mmHg [1,2], and some prospective clinical studies have also shown better outcomes when CPP was maintained above 70 mmHg [3-6]] compared to the 40% mortality rate reported for the Traumatic Coma Databank patients [7]. More recent prospective studies, however, found no differences in outcome when CPP was maintained above 50 mmHg [8] or 60 mmHg [9] and warn about a higher risk of acute respiratory distress syndrome (ARDS) with a CPP above 70 mmHg [8].

There is also controversy about the advised CPP threshold to ensure proper tissue oxygen delivery and variable results have been reported in the literature. Some authors found a relationship between CPP and PtiO_2 _focusing on values under a threshold of 60 mmHg [10]. It has also been shown that an increase of CPP from 32 to 67 mmHg significantly improved brain tissue pO_2 _[11] but that further increases in the CPP did not improve it [11,12]. Moreover, a zone of intact PtiO_2 _autoregulation with a CPP between 70 and 90 mmHg [13] has also been shown. Nevertheless, other authors observed that increasing CPP into 'supranormal values' was helpful in normalizing PtiO_2 _in ischemic areas [14], and others have also reported a positive correlation between CPP and PtiO_2_, with a peak of PtiO_2 _at a CPP value around 78 mmHg [15].

The importance of CPP for maintaining an adequate level of tissue oxygenation is a point of clinical relevance. Taking into consideration all these controversies, our objective was to study the distribution of PtiO_2 _samples assumed hypoxic at different CPP thresholds, using prospective multimodality monitoring in a series of patients with severe head injuries in order to further refine the optimal CPP based on this newer monitoring technique.

## Materials and methods

### Patient selection and profile

Over a one year period, 24 consecutive patients with TBI and a post-resuscitation Glasgow Coma Score (GCS) < 9 were initially enrolled in this study. As an observational study, approval from the local Research and Ethics Committee or written informed consent was deemed unnecessary. The scoring systems used for quantifying the severity of the illness of the patients were the Acute Physiology and Chronic Health Evaluation (APACHE) II score, the Revised Trauma Score for triage (T-RTS) and the Injury Severity Score (ISS). The Traumatic Coma Databank computed tomography (CT) scan classification was used to categorize the head injury severity of patients, and the patient outcome was scored according to the Glasgow Outcome Scale (GOS).

### Continuous monitoring and sampling of multimodal physiological data

Intracranial pressure (ICP) and PtiO_2 _were continuously monitored using an intraparenchymal probe (ICP transducer Ventrix, Integra Neurosciences, Plainsboro, New Jersey, USA), epidural probe (Spiegelberg GmHB & Co., Hamburg, Germany) or ventricular catheter, and a flexible polarographic Clark-type O2 probe (Licox GMS mbH, Kiel, Germany), respectively. If intraparenchymal probes were used, PtiO_2 _and ICP probes were inserted through a unique screw into the frontal lobe to monitor water shade territory between anterior and middle cerebral arteries of uninjured areas, according to CT. Electrocardiogram, invasive mean arterial blood pressure, peripheral arterial oxygen saturation, and end-tidal carbon dioxide were also continuously monitored (Marquette Solar 8000 M, GE Medical Systems, Bradford, West Yorkshire, UK). Hourly snapshot values of all these parameters were sampled for offline analysis. Exclusion criteria for taking into account multimodal data samples for analysis were implemented as follows, with the aim of reducing confounding factors in the study of PtiO_2_-CPP relationships. Firstly, if initial PtiO_2 _values were low and unstable (< 10 mmHg but rising), early multimodal samples of each patient were rejected until PtiO_2 _values had reached a plateau phase in the time axis to avoid artifactual PtiO_2 _changes related to known run-in time errors linked to micro-injuries post-catheter insertion or frequent low PtiO_2 _readings during the first 24 h [16]. Lastly, multimodal samples that had ultra-low end-tidal carbon dioxide values (below 25 mmHg, which usually corresponded to pCO_2 _values of less than 30 mmHg) were, by agreement, discarded with the intention of minimizing hypoxic PtiO_2 _changes acutely related to this variable [17]. Several blood tests (usually two or more) were made daily to check if physiological variables remained stable in order to identify and correct causes of non-ischemic hypoxia [18]. The principal causes of low-extractivity hypoxia, such as anemia and hypoxemia, and the causes of high-affinity hypoxia (e.g. hypocapnia with respiratory alkalosis, hypothermia) were avoided, with the intention of maintaining an arterial oxygen pressure (PaO_2_) greater than 100 mmHg, an arterial carbon dioxide pressure (PaCO_2_) around 35 mmHg, a hematocrit level above 30% and a body temperature of 36 to 38°C.

### Routine treatment

All patients were treated following a standardized protocol consistent with the *Guidelines for the Management of Severe Traumatic Brain Injury *[19], which included control of body temperature, elevation of the head of the bed, seizure prophylaxis, avoidance of jugular outflow obstruction, sedation, intubation, mechanical ventilation and complete volume resuscitation to maintain a CPP of 60 to 70 mmHg or more. Space-occupying lesions larger than 25 cm^3^ were surgically removed. When the ICP exceeded 20 mmHg, therapeutic interventions were initiated step by step, inducing external ventricular drainage when possible, moderate hypocapnia (PaCO_2 _30 to 35 mmHg), deeper sedation and muscle relaxation, and relative hyperosmolarity with mannitol or hypertonic NaCl infusions. When ICP was not under control despite these therapeutic modalities, second tier therapies were considered: arterial hypertension with noradrenaline, hyperventilation to PaCO_2 _< 30 mmHg with PtiO_2 _monitoring and high dose barbiturate therapy. Besides this protocol, if PtiO_2 _data were below 15 mmHg, and once artifactual measurements and causes of non-ischemic hypoxia were ruled out [18], CPP was augmented above 70 mmHg and higher values with noradrenaline to improve it.

### Data analysis

For analytical purposes, 15 mmHg was elected as the ischemic threshold [16,20] and hypoxic PtiO_2 _samples (or at risk of hypoxia) were defined when PtiO_2 _was below or equal to this threshold. Three CPP thresholds were also chosen to study the percentage of hypoxic PtiO_2 _samples at different CPP intervals, according to what could be considered values of insufficient (< 60 mmHg), advised (60 to 70 mmHg) and excessive (> 70 mm Hg) CPP in the absence of brain ischemia, following updated guidelines for CPP management of severe traumatic brain injury (copyrighted by the Brain Trauma Foundation) [21]. The Kolmogorov-Smirnov test was applied to verify if the variables followed a normal distribution. When the variables were found not to be normally distributed, comparisons between groups of data were made using Kruskal-Wallis H and Mann-Whitney U tests to detect differences in the distribution of samples, and Spearman's Rho coefficient to assess the relationship between two quantitative variables. As visual representations of data, box-and-whisker plots were chosen for handling many values due to their ability to show only certain statistics (the median, the lower quartile and the upper quartile, and the lowest and highest values in the distribution of a given set of data) rather than all the data distribution. SPSS 12.0S for windows (SPSS Inc, Chicago, Illinois, USA) was the computer software used for statistical analysis of the data.

## Results

### Patient characteristics

Of 24 patients initially enrolled, two were not included in the study because of technical problems with the PtiO_2 _monitoring: one patient developed a small hematoma around the tip of the PtiO_2 _catheter and another had the PtiO_2 _catheter positioned in the subarachnoid space. The characteristics of the remaining 22 patients were as follows. Their age was 30 ± 12 years, and 18 of the patients were male. The causal mechanism was road traffic accident in fifteen cases, fall in six cases and aggression in one case. The median post-resuscitation Glasgow Coma Score was 6. The mean APACHE II score was 17 ± 4, the Revised Trauma Score 9 ± 1, and the Injury Severity Score 31 ± 7. According to the Traumatic Coma Databank classification, eight were class II (diffuse injury), seven class III (diffuse injury with swelling), six class V (mass lesion surgically evacuated) and one class VI (mass lesion not operated).

Following the American-European consensus conference on ARDS [22], only one patient developed ARDS (4.5%), in the context of multi-organ failure, and this complication had no fatal consequences. The outcome according to the GOS at the neurosurgical critical care unit was: three dead (GOS 1, 14%), one vegetative (GOS 2, 0%), thirteen severely disabled (GOS 3, 59%), four moderately disabled (GOS 4, 18%), and one good recovery (GOS 5, 5%). Follow up was not possible in two cases, and the outcome according to the GOS of the remaining twenty patients after twelve months was as follows: three dead (GOS 1, 15%), none vegetative (GOS 2, 0%), eight severely disabled (GOS 3, 40%), four moderately disabled (GOS 4, 20%), five good recovery (GOS 5, 25%). The outcome seemed to be better in those patients without hypoxic samples (GOS 4 and 5 after twelve months, 55% versus 36%), although this finding did not reach statistical significance.

### PtiO_2_-CPP relationship

After the multimodal determinations that fulfilled the exclusion criteria were discarded, 1,672 hourly snapshot samples from 22 patients were analyzed. The main physiological data during the course of the study are shown in Table 1. Low CPP values (< 60 mm Hg) were due to high ICP values (≥ 20 mm Hg) rather than low mean arterial blood pressure data in the vast majority of samples (> 90%).

**Figure 1:**
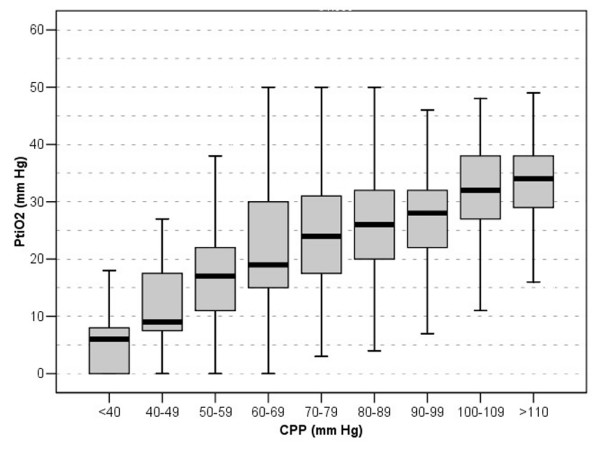
Brain tissue oxygen pressure (PtiO_2_) versus cerebral perfusion pressure (CPP). This box-and-whisker plot shows the relationship between PtiO_2 _and CPP. The median, the lower quartile and the upper quartile, and the lowest and highest values in the distribution of samples (N = 1,672 hourly snapshot samples) are represented by the black horizontal bar, the upper and lower end of each box, and the upper and lower end of its error bars, respectively.

To study the PtiO_2_-CPP relationship, the method of analysis was as follows. Firstly, PtiO_2 _was plotted versus CPP in a box-and-whisker plot, grouping CPP values in intervals of 10 mmHg. The course of this plot shows that cerebral oxygenation is directly related to cerebral perfusion and even more closely to low CPP values with a breakpoint around 60 to 70 mmHg (Fig. 1). Secondly, the correlation between PtiO_2 _and CPP variables was analyzed. As the Kolmogorov-Smirnov Z test showed that the PtiO_2 _variable did not follow a normal distribution (Z = 3.24), a Spearman's Rho coefficient was calculated for values grouped in intervals below different CPP thresholds (Table 2). There was a statistically significant correlation between PtiO_2 _and CPP that was more powerful for CPP values below 60 mmHg (Spearman's Rho coefficient 0.50, p < 0.01) than for others (Spearman's Rho coefficient around 0.2, p < 0.01).

### CPP thresholds for hypoxia

**Figure 2:**
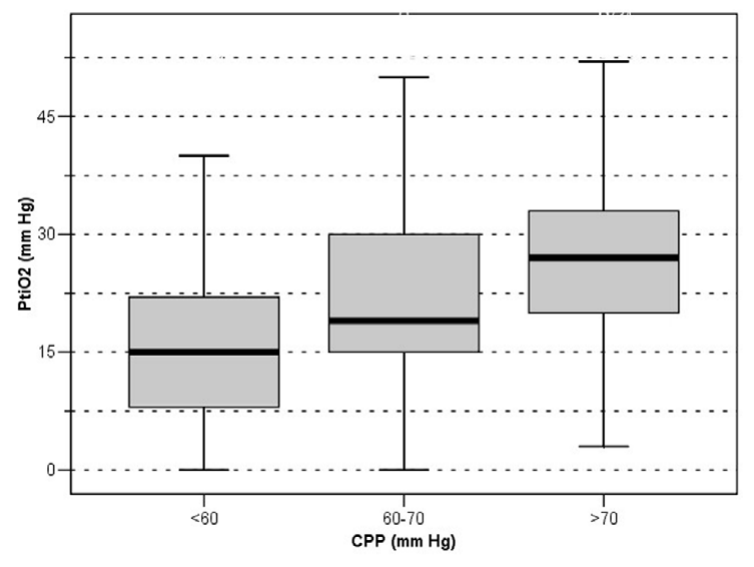
Brain tissue oxygen pressure (PtiO_2_) versus different cerebral perfusion pressure (CPP) thresholds. This box-and-whisker plot shows the PtiO_2_-CPP relationship at different CPP thresholds. Half of samples were hypoxic (PtiO_2 _≤ 15 mmHg) for CPP values below 60, and were reduced to a quarter and 10% for CPP values between 60 and 70 mmHg, and above 70 mmHg, respectively (p < 0.01).

The distribution of PtiO_2 _samples at different thresholds was calculated for each interval and represented by a box-and-whisker plot (Fig. 2). This distribution showed a higher percentage of hypoxic samples at lower CPP thresholds: when CPP was below 60 mmHg (which occurred in 55% of patients), half of the PtiO_2 _samples were hypoxic; if CPP was between 60 and 70 mmHg (86% of patients), a quarter of PtiO_2 _samples were still hypoxic; but when CPP was above 70 mmHg (100% of patients), only 10% of PtiO_2 _measurements sampled were in the hypoxia range. The Kruskal-Wallis and Mann-Whitney tests confirmed that the differences observed in the distribution of PtiO_2 _samples in relation to CPP thresholds were statistically significant (p < 0.01). PtiO_2 _percentiles calculated for these CPP thresholds are shown in Table 3.

## Discussion

Our main results demonstrate, firstly, that the PtiO_2 _data measured in uninjured areas of brain tissue of severe TBI patients increases with higher CPP. Secondly, the PtiO_2_-CPP relationship shows a lower breakpoint between 60 and 70 mmHg, indicating a stronger dependence below this autoregulatory threshold. Lastly, a CPP above 70 mmHg could be necessary to reduce the number of hypoxic PtiO_2 _samples by less than half (from 25% to 10%, p < 0.01).

Although the last update of the Guidelines for the Management of Severe Traumatic Brain Injury and Cerebral Perfusion Pressure (copyrighted by the Brain Trauma Foundation) recommends that "CPP should be maintained at a minimum of 60 mmHg, and in the absence of cerebral ischemia, aggressive attempts to maintain CPP above 70 mmHg with fluids and pressors should be avoided because of the risk of adult respiratory distress syndrome" [21], our study highlights that the risk of tissue hypoxia could be really high when CPP is below the threshold of 60 mmHg but still elevated when CPP is slightly over this threshold.

### The effect of CPP on PtiO_2_

Our data supports an additional improvement in PtiO_2 _by a further elevation of CPP in all the intervals studied: below 60 mmHg, between 60 and 70 mmHg and above 70 mmHg. This is a different conclusion from that drawn by other authors who demonstrated an improvement in cerebral oxygenation when the CPP increased from 32 to 67 mmHg but not when the CPP increased from 68 to 84 mmHg [11]. Several reasons may explain these apparently contradictory findings. First of these may be the different study designs used, as we did not carry out a trial but observed the effect of both spontaneous and induced increases of CPP on PtiO_2_. Second is the higher number of samples analysed, which could help to reach statistical significance. Last is the different vasoactive drugs used to augment CPP (noradrenaline versus dopamine), as there is increasing evidence that the vasoactive agent used may be of importance for brain oxygenation and the response may be more or less predictable [23,24].

It has been confirmed that PtiO_2 _is highly dependent on CPP in ischemic areas and that a 'supra-normal' CPP invariably improves PtiO_2_, with an even greater improvement when PtiO_2 _is low [14]. Our study also supports this hypothesis when PtiO_2 _was measured in apparent non-ischemic areas, defined as normal density areas in CT scans. As a recent study using positron emission tomography (PET) imaging suggests [25], "apparent normal areas" found in CT could in fact be zones where the autoregulation may be disturbed and shifted to the right and, perhaps, the normality on radiograph CT may not reliably predict the PtiO_2 _response to CPP augmentation.

A PtiO_2_-CPP relationship with a plateau phase in cerebral tissue oxygenation for CPP values between 70 and 90 mmHg, similar to that known to be present in cerebral blood flow velocity, has been demonstrated recently, which suggests a close link between cerebral blood flow autoregulation and cerebral tissue oxygen reactivity [13]. Although the course followed by the PtiO_2_-CPP relationship in our study remained as reported, we observed a statistically significant increase in PtiO_2 _with CPP above 70 mmHg. These results are in accordance with a later study that assessed the effects of CPP augmentation on regional physiology and metabolism in TBI patients using ^15^O PET, brain tissue oxygen monitoring, and cerebral microdialysis [25]. This study shows that CPP augmentation from 70 mmHg to 90 mmHg significantly increased levels of brain tissue oxygen and reduced the regional oxygen extraction fraction, although these changes did not translate into predictable changes in regional chemistry [25]. Perhaps additional data are needed to clearly demonstrate that PtiO_2 _does not change within the range of supposed autoregulation.

On the other hand, despite inducing augmentation of CPP with fluids and vasoactive drugs as part of our protocol management, the frequency of ARDS found in our study could be considered low (4.5%) in relation to other reports [26] that use the same ARDS definition [22]. Furthermore, the impact of ARDS on mortality was nil, regardless of whether we induced CPP values noticeably above 70 mmHg to improve CPP and cerebral oxygenation if needed.

### Limitations of the study

Hypoxia is not synonymous to ischemia and, as described by Siggard-Andersen *et al*. [18], many factors apart from blood flow may influence tissue oxygen availability, including the total concentration of O_2 _in blood (which depends on the PaO_2 _and the concentration of effective Hemoglobin (Hb)), the affinity of the Hb (which also depends on other factors such as temperature, pH, and the concentration of 2,3-Diphosphoglycerate (DPG)), the degree of arterio-venous shunt, the diffusion of O_2 _from the capillary, and the metabolic rate of oxygen consumption, among others. Determining the importance of CPP on PtiO_2 _among all the variables that may influence the tissue oxygen availability in a heterogeneous group of head injured patients may be a difficult task. In this context, efforts have been made to stabilize all those variables not related to cerebral perfusion that may modify the availability of oxygen and to analyze only those samples that did not fulfill the mentioned exclusion criteria, to avoid non-ischemic causes of hypoxia.

Regional differences in brain perfusion, metabolism and oxygenation could be large among different brain areas in TBI patients, and PtiO_2 _monitoring as a regional technique only measures tissue oxygen pressure in a very small tissue volume. Although the addition of jugular venous oxygen saturation monitoring could provide some insight into the global 'oxygenation' of the brain, this technique was not routinely applied because of its cumbersome handling and poor quality data [27], despite its convincing scientific background. The use of PtiO_2 _measurements in non-lesioned tissue, however, was systematically applied as a strategy to reflect how systemic factors may influence brain tissue oxygenation because of the reliability of the method and its safety [20].

Although thresholds for brain hypoxia vary widely according to the different study approaches used in the literature [11,16,20], a recent study using PET suggests that the ischemic threshold may lie below 14 mmHg [25], and the mild tissue hypoxia threshold could be considered to be around 15 mmHg following several outcome studies [16,20]. As we decided to use 15 mmHg as the hypoxia threshold in order to reach valid conclusions for all degrees of hypoxia (mild, moderate or severe), our data analysis may be less specific but more sensitive for actual hypoxia.

The empiric augmentation of CPP to higher levels to improve brain tissue oxygenation and minimize hypoxic events is a simplistic approach. Besides being unnecessary and even dangerous [26], it is not supported by the study as it did not prospectively analyze the effect of different CPP augmentation regimens on brain PtiO_2_.

Although the percentage of patients with GOS 4 and 5 after 12 months was higher in those who did not have hypoxic events, this apparent better outcome did not reach statistical significance, probably because of the small size of the sample (N = 20).

## Conclusion

Our study highlights that the risk of brain tissue hypoxia could be really high when CPP is below the normally recommended threshold of 60 mmHg but still elevated when it is slightly over this threshold, and clearly demonstrates that brain tissue hypoxia occurs less frequently at higher CPP (p < 0.01). Although there is concern about aggressively maintaining CPP above 60 mmHg in the absence of cerebral ischemia due to the risk of ARDS, maintaining CPP above 60 and 70 mmHg may be of practical relevance as a strategy for improving cerebral perfusion and cerebral oxygenation in cases of proven brain hypoxia once other causes of non-ischemic hypoxia have been ruled out, as it decreases the risk of cerebral hypoxia in severe TBI.

## Key messages

• 	In severe TBI, the risk of brain tissue hypoxia is common when CPP is below 60 mmHg, less frequent when it is between 60 and 70 mmHg, and lower above these CPP thresholds.

• 	Brain hypoxia occurs less frequently at higher CPP

• 	In the case of proven brain hypoxia and once treatable non-ischemic causes have been stabilized, maintaining higher CPP may be helpful.

## Abbreviations

APACHE = acute physiology and chronic health evaluation; ARDS = acute respiratory distress syndrome; CPP = cerebral perfusion pressure; CT = computed tomography; GCS = Glasgow coma score; GOS = glasgow outcome scale; ICP = intracranial pressure; ISS = injury severity score; PaCO_2 _= arterial carbon dioxide pressure;PaO_2_ = arterial oxygen pressure; PET = positron emission tomography; PtiO_2_ = tissue oxygen pressure; TBI = traumatic brain injury; T-RTS = revised trauma score for triage.

## Competing interests

The authors declare that they have no competing interests.

## Authors' contributions

AJM carried out the design of the study, data collection, analysis, interpretation of data and writing of the manuscript. FM actively participated in the conception and design of the study, interpretation of data and drafting the manuscript. AC assisted in the design of the study and performed the statistical analysis. JMD made substantial contributions to interpretation of data and critical revision of the manuscript. MDR contributed to acquisition of data. JV carried out the neurosurgical support of multimodal monitoring. JMF and MAM participated in coordinating the study and revising the manuscript critically. The authors have given final approval of the version to be published

## Figures and Tables

**Table 1:** Descriptive statistics of continuously monitored physiological data

Variable	Minimum	Maximum	Mean	Standard deviation
Mean arterial blood pressure (mmHg)	62	139	96	11
Intracranial pressure (mmHg)	0	69	15	10
Cerebral perfusion pressure (mmHg)	27	128	81	14
End tidal carbon dioxide (mmHg)	25	64	32	4
Peripheral saturation of oxygen (%)	78	100	99	2
Temperature (°C)	31	39	36.7	0.9
Hemoglobin (g/dl)	6.7	14	10.2	1.4
Hours monitored per patient	27	195	40	36

**Table 2:** Correlation between tissue oxygen pressure and cerebral perfusion pressure below different cerebral perfusion pressure thresholds

		CPP < 50 mmHg	CPP < 60 mmHg	CPP < 70 mmHg	CPP < 80 mmHg	CPP < 90 mmHg	CPP < 100 mmHg	CPP < 130 mmHg
PtiO_2_	Spearman's Rho coefficient^a^	0.54	0.50	0.19	0.24	0.24	0.24	0.29
	Probability	< 0.05	< 0.01	< 0.01	< 0.01	< 0.01	< 0.01	< 0.01
	N samples	16	75	308	776	1,252	1,510	1,672

^a^Bilateral correlation. CPP, cerebral perfusion pressure; PtiO_2_, tissue oxygen pressure.

**Table 3:** Percentiles of tissue oxygen pressure samples at different cerebral perfusion pressure thresholds

	PtiO_2 _percentiles (mmHg)						
	5	10	25	50	75	90	95
CPP < 60 mmHg	0	4	8	15^a^	22	35	42
CPP 60–70 mmHg	8	10	15^a^	19	30	48	51
CPP > 70 mmHg	12	15^a^	20	27	33	41	48

^a^Hypoxia threshold. CPP, cerebral perfusion pressure; PtiO_2_, tissue oxygen pressure.
